# Validation of a Low Dose Simulation Technique for Computed Tomography Images

**DOI:** 10.1371/journal.pone.0107843

**Published:** 2014-09-23

**Authors:** Daniela Muenzel, Thomas Koehler, Kevin Brown, Stanislav Žabić, Alexander A. Fingerle, Simone Waldt, Edgar Bendik, Tina Zahel, Armin Schneider, Martin Dobritz, Ernst J. Rummeny, Peter B. Noël

**Affiliations:** 1 Department of Radiology, Technische Universitaet Muenchen, Munich, Germany; 2 Philips Technologie GmbH, Innovative Technologies, Hamburg, Germany; 3 Philips Healthcare, Cleveland, Ohio, United States of America; 4 MITI - Minimal-invasive Interdisciplinary therapeutic intervention research group, Technische Universitaet Muenchen, Munich, Germany; University of Groningen, University Medical Center Groningen, Netherlands

## Abstract

**Purpose:**

Evaluation of a new software tool for generation of simulated low-dose computed tomography (CT) images from an original higher dose scan.

**Materials and Methods:**

Original CT scan data (100 mAs, 80 mAs, 60 mAs, 40 mAs, 20 mAs, 10 mAs; 100 kV) of a swine were acquired (approved by the regional governmental commission for animal protection). Simulations of CT acquisition with a lower dose (simulated 10–80 mAs) were calculated using a low-dose simulation algorithm. The simulations were compared to the originals of the same dose level with regard to density values and image noise. Four radiologists assessed the realistic visual appearance of the simulated images.

**Results:**

Image characteristics of simulated low dose scans were similar to the originals. Mean overall discrepancy of image noise and CT values was −1.2% (range −9% to 3.2%) and −0.2% (range −8.2% to 3.2%), respectively, p>0.05. Confidence intervals of discrepancies ranged between 0.9–10.2 HU (noise) and 1.9–13.4 HU (CT values), without significant differences (p>0.05). Subjective observer evaluation of image appearance showed no visually detectable difference.

**Conclusion:**

Simulated low dose images showed excellent agreement with the originals concerning image noise, CT density values, and subjective assessment of the visual appearance of the simulated images. An authentic low-dose simulation opens up opportunity with regard to staff education, protocol optimization and introduction of new techniques.

## Introduction

Computed tomography (CT) examination plays a fundamental role in an all-day radiological work-up of patients in hospitals with modern healthcare equipment all around the world. Its excellent diagnostic value combined with a very short image acquisition time makes it a basic and essential diagnostic imaging tool. During the last decade there were a lot of discussions concerning an increased risk of cancer caused by the use of ionizing radiation in medicine [1], [2]. On the other hand, Hendee and O’Connor recently warned against an anxiety and fear of patients sensationalized by public media with the risk of delayed or refused medical imaging and, as a consequence, delayed or missed diagnosis [3]. A clinically justified CT examination and its benefit of an accurate diagnostic work-up always outweigh its associated individual risks like e.g. stochastically induced risk of cancer [4], [5]. However, these considerations encourage the demand for establishment of CT examination protocols according to the “as low as reasonably achievable” (ALARA) principle. This means an image acquisition at a radiation dose as low as possible while still maintaining a diagnostic image quality. A valid determination of the optimized dose levels for all specific CT examination protocols would demand a comparison of images of patients obtained at different dose levels. However, this would require repeated scans of the patients resulting in a significant increase of radiation exposure to these patients or probands. Therefore, it is desirable to have a computer simulation tool for reconstructing images from one original data set simulating images were acquired at lower dose levels.

The aim of this study was to evaluate and validate the software tool described in [6] for simulation of a lower dose CT acquisition from an original higher dose scan, using an animal model for non-contrast and contrast enhanced CT scans.

## Methods

### Animal experiment

A female landrace pig was examined with a bodyweight of 49 kg. CT image acquisition was performed with the animal under deep general anaesthesia with endotracheal intubation and controlled ventilation. All animal procedures were performed in strict accordance with the German animal protection law and were approved by the Regierung von Oberbayern; 209.1/211-2531.3-5/03.

The animal received regular feeding until 24 h before the procedure. Subsequently, it had a liquid diet until 12 h before the intervention and was kept off food for the remaining time. An 18 G venous access was placed in an ear vein for the administration of iodinated contrast. Pre-anaesthesia sedation was performed with an intramuscular injection of Azaperon (2.0 mg/kg), Atropin (0.02 mg/kg) and Ketamin (15 mg/kg). General anaesthesia was initiated by the injection of Propofol (1%) by effect. After endotracheal intubation maintenance of anaesthesia was achieved by continuous injection of propofol 2% with bolus application of Fentanyl. Oxygenation, temperature, and heart rate were continuously monitored and anaesthetic medication adapted if necessary. After completion of the CT scans, the pig was euthanized using a lethal dose of pentobarbitone and potassium chloride.

### CT image acquisition

CT examinations were performed using a wide coverage 256-slice multidetector CT scanner (Brilliance iCT, Philips Healthcare, Cleveland, OH, USA). The animal was positioned in the center of the gantry. Spiral data acquisition was performed using 64×0.625 mm collimation, a pitch factor of 0.985, and a gantry rotation time of 0.4 s. Tube settings were 100 kV in all studies and 100 mAs, 80 mAs, 60 mAs, 40 mAs, 20 mAs, and 10 mAs, respectively.

**Figure 1:**
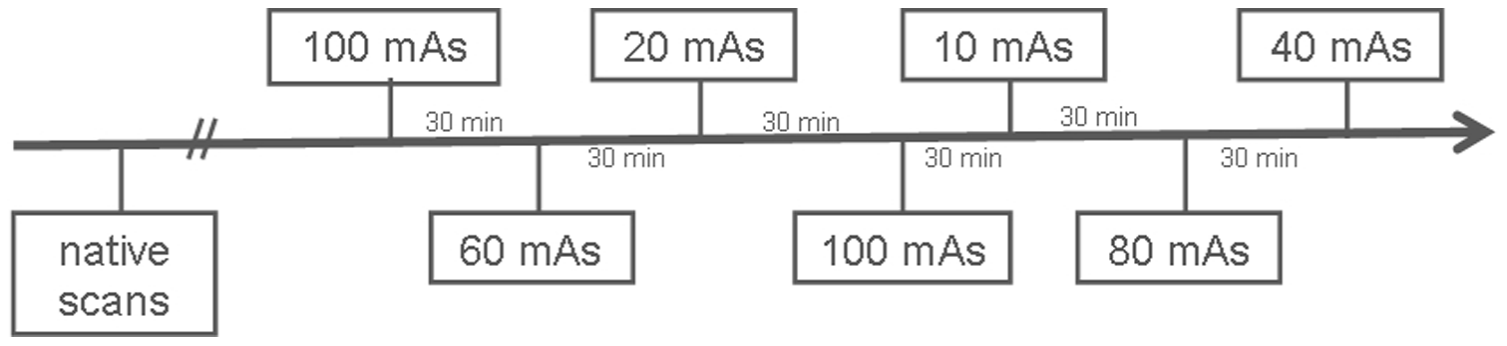
Time flow of CT scans at different dose levels. First, all native scans were performed. Afterwards, contrast enhanced scans were acquired, in a random order of the different dose levels. There was a gap of 30 minutes between the scans. 100 mAs scan ( =  base of the simulations) was achieved twice in order to minimize discrepancies between originals and simulations caused by differences in contrast accumulation.

Native and contrast enhanced CT examination of the chest and abdomen in arterial contrast phase were performed during end-expiratory breath-hold with the pig in supine position. For the contrast enhanced scans, a fixed volume of 50 ml of contrast agent (Imeron 400 MCT, Bracco Imaging Deutschland GmbH, Konstanz, Germany) was injected at a flow of 4 ml/s into an ear vein via an 18-gauge catheter using a dual syringe injection system (Stellant, MEDRAD, Inc., Indianola, Pennsylvania). The contrast bolus was followed by 40 mL saline solution. The scanner started data acquisition by bolus tracking. The contrast agent was washed out between the contrast-enhanced CT examinations by saline flushing for 30 minutes. However, there was an accumulation of contrast load over time, with a subsequent increase of HU of the liver parenchyma from 68 HU to 141 HU (100 mAs: 68 HU, 60 mAs: 82 HU, 20 mAs: 90 HU, 100 mAs: 116 HU, 10 mAs: 113 HU, 80 mAs: 125 HU, 40 mAs: 142 HU). To avoid any unblinding due to insufficient contrast wash-out or accumulation of contrast material in the urinary tract and the organs, CT scans of different dose levels were performed in a random order. In addition, 100 mAs data set (base of all simulations) was scanned twice, once at the beginning and once in the middle of the study protocol, in order to create simulations with more and less contrast material in the urinary tract. The time flow of image acquisition is illustrated in Figure 1.

Standard image reconstructions (filtered back projection) were obtained with 3 mm slice thickness using the CA (smooth) kernel. The reconstruction field of view was 380 mm and matrix size was 512×512.

### CT low dose simulation

The CT raw data of the 100 mAs scans were retrieved from the scanner and used as input for the low dose simulation tool, which takes in account both photonic and electronic noise. Details of the low dose simulation algorithm are given in reference [6]. The resulting simulated scans at target mAs ranging from 80 mAs down to 10 mAs were reconstructed off-line with the same reconstruction parameters as the original scans.

### CT values and image noise

Hounsfield units (HU) of defined regions of interest (ROI) were determined and compared for original and simulated data. Image noise was defined as the standard deviation of a 50 mm^2^ ROI. Therefore, 10 representative ROIs were defined for the following material: back muscles, subcutaneous fat tissue of the ventral abdominal wall, lung tissue, fluid content of the gallbladder, and the lumbar vertebral bodies of the spine. For comparison of noise levels in different anatomical regions, image noise was defined of the shoulder girdle, dorsum, abdominal wall, and pelvis. Results of image noise were statistically analyzed for original and simulated data at corresponding radiation dose levels. Confidence intervals of mean discrepancies between the originals and simulations were calculated for all tissue and dose levels, respectively.

### Observer discrimination of simulated versus original images

For a qualitative assessment of the simulated images and to approve a realistic appearance of the images, observer evaluation was performed for the contrast- enhanced images of the swine. Therefore, transverse and coronal slices (slice thickness 3 mm) were formatted for the original and simulated data sets for all dose levels. Selection of imaging features and parameters are shown in Table 1. Therefore, multiplanar reformations with different windows settings were created. In total, 160 images (5 dose levels, 16 reconstructions, original and simulation) were evaluated. 2D images were randomly arranged one by one for subjective image evaluation. Four experienced radiologists (mean clinical experience 7 years, range 3–15 years) were instructed to rate each image with regard to originality (1 =  original scan or 2 =  simulated one).

**Table 1:** Synopsis of the images created for individual image assessment (original versus simulated).

No.	Slice orientation	Location	Main tissue
1	transverse	Aortic arch	Vessel, lung
2	transverse	mediastinum	heart
3	transverse	hilus	Mediastinum, vessel, lung
4	transverse	abdomen	liver, stomach, spleen
5	transverse	abdomen	gallbladder, liver
6	transverse	abdomen	small bowel
7	transverse	abdomen	kidneys, small bowel
8	transverse	pelvis	bladder, bowel, soft tissue
9	transverse	pelvis	bone
10	transverse	chest	lung
11	coronal	chest	lung
12	coronal	mediastinum	Mediastinum, vessel, lung
13	coronal	abdomen	Liver, spleen, bowel, kidneys
14	coronal	abdomen	Aorta, retroperitoneum
15	coronal	chest	ribs
16	Sagittal	spine	bone

Typical clinical reformations of characteristic anatomic regions with appropriate windows settings were created, with a total of 16 images prepared for all dose levels.

### Statistical Analysis

Continuous data are expressed as arithmetic mean ± SD. Differences of the mean are displayed with confidence intervals. A two-tailed paired Student t-test was performed for comparison of image noise and Hounsfield units of original and simulated images for different regions of interest. Cohen kappa statistic was used for evaluation of interobserver agreement. A p-value ≤0.05 was considered to indicate statistical significance. All statistics were computed with Microsoft Excel and SPSS.

## Results

### CT values and image noise

CTDI values of all scan data (100 kV; 100 mAs, 80 mAs, 60 mAs, 40 mAs, 20 mAs, and 10 mAs, respectively) ranged from 4.4 mGy to 0.44 mGy.

**Figure 2:**
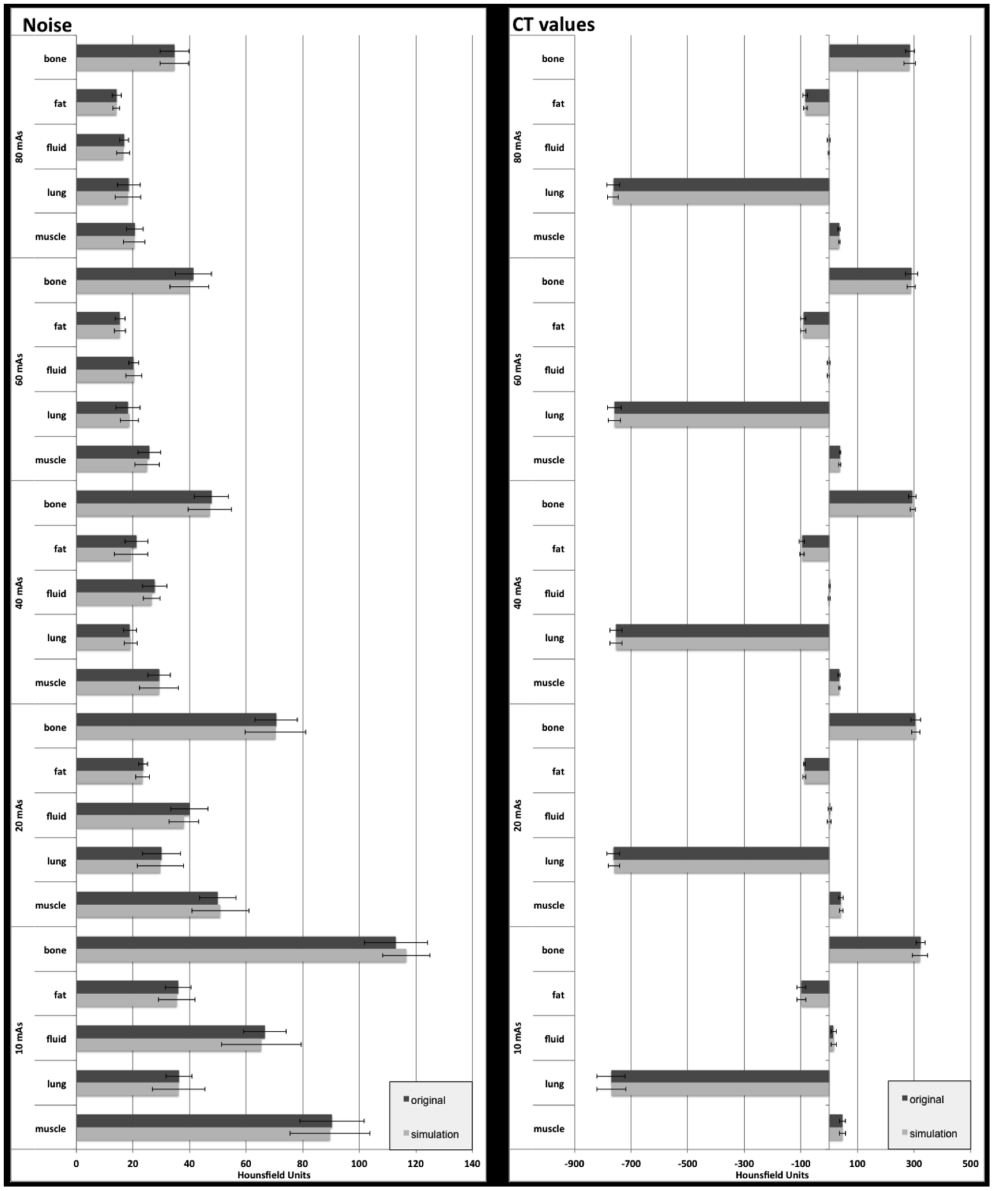
Mean values of image noise (left side) and CT values (right side) of characteristic tissues (bone, fat, fluid, lung, and muscle) for original (black bars) and simulated (grey bars) transverse slice images (3 mm slice thickness) at different dose levels (10, 20, 40, 60, and 80 mAs). There were no significant differences with mean discrepancies of −1.2% in image noise and −0.2% in CT values between simulated and original images. Error bars indicate standard deviation.

Mean density values of different tissues such as soft tissue, bone, lung, fluid, and fat were determined in characteristic slices of the non-enhanced CT examination of the animal study. Corresponding mean HU values are shown in Figure 2.

Mean discrepancy of image noise between original and simulated CT images calculated for all tissues and all dose levels was −1.2% (range −9% to 3.2%; p>0.05). The differences in CT values between original and simulated data ranged between −8.2% and 3.2%, with a mean of −0.2% (p>0.05). Image noise and CT values of characteristic tissue at different dose levels are presented in Figure 2. Similar noise was obtained for all dose levels in original and simulated images, with mean values of 21.0 vs. 20.8 (80 mAs), 24.2 vs. 23.9 (60 mAs), 29.0 vs. 28.3 (40 mAs), 42.9 vs. 42.5 (20 mAs), and 68.4 vs. 68.7 (10 mAs), p>0.05, respectively. Image noise in different anatomical regions is shown in Table 2.

**Table 2:** Image noise for different anatomical regions in original and simulated images at different dose levels.

		dose level (mAs)				
anatomy		80	60	40	20	10
shoulder girdle	original	36.4	38.2	68.7	117.6	174.3
	simulation	38.7	40.2	68.7	115.0	173.2
dorsum	original	27.0	27.9	36.6	57.2	108.3
	simulation	27.1	28.8	33.7	58.8	109.9
abdominal wall	original	20.4	24.2	29.5	32.2	66.3
	simulation	20.5	25.5	28.7	36.2	65.3
pelvis	original	70.3	76.7	94.1	134.0	206.3
	simulation	68.7	77.5	98.5	130.4	204.5

There were no significant differences between originals and simulations (p>0.05).

**Figure 3:**
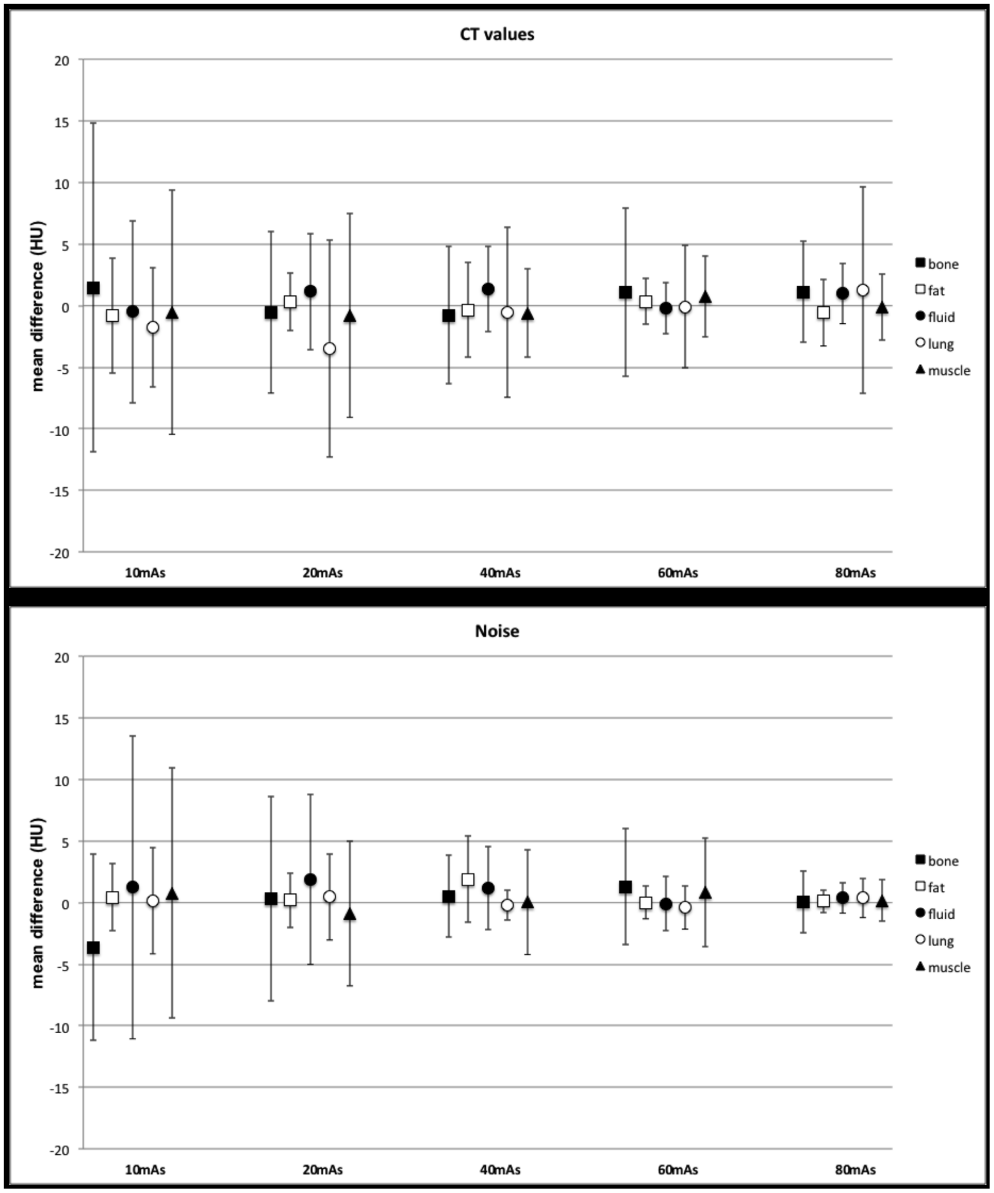
Differences of the mean and confidence interval for noise (a) and CT values (b) for bone, fat, fluid, lung, and muscle at different dose levels.

Differences of the mean of noise and CT value measurements for original and simulated images were calculated for each tissue at all dose levels (Figure 3). Confidence intervals for all tissues and dose levels ranged between 0.9–10.2 HU (noise) and 1.9–13.4 HU (CT values). The value of 0 was included in all confidence intervals, and there were no significant differences between the original and simulations for all tissue and all dose levels (p>0.05).

### Observer discrimination of simulated versus original images

**Figure 4:**
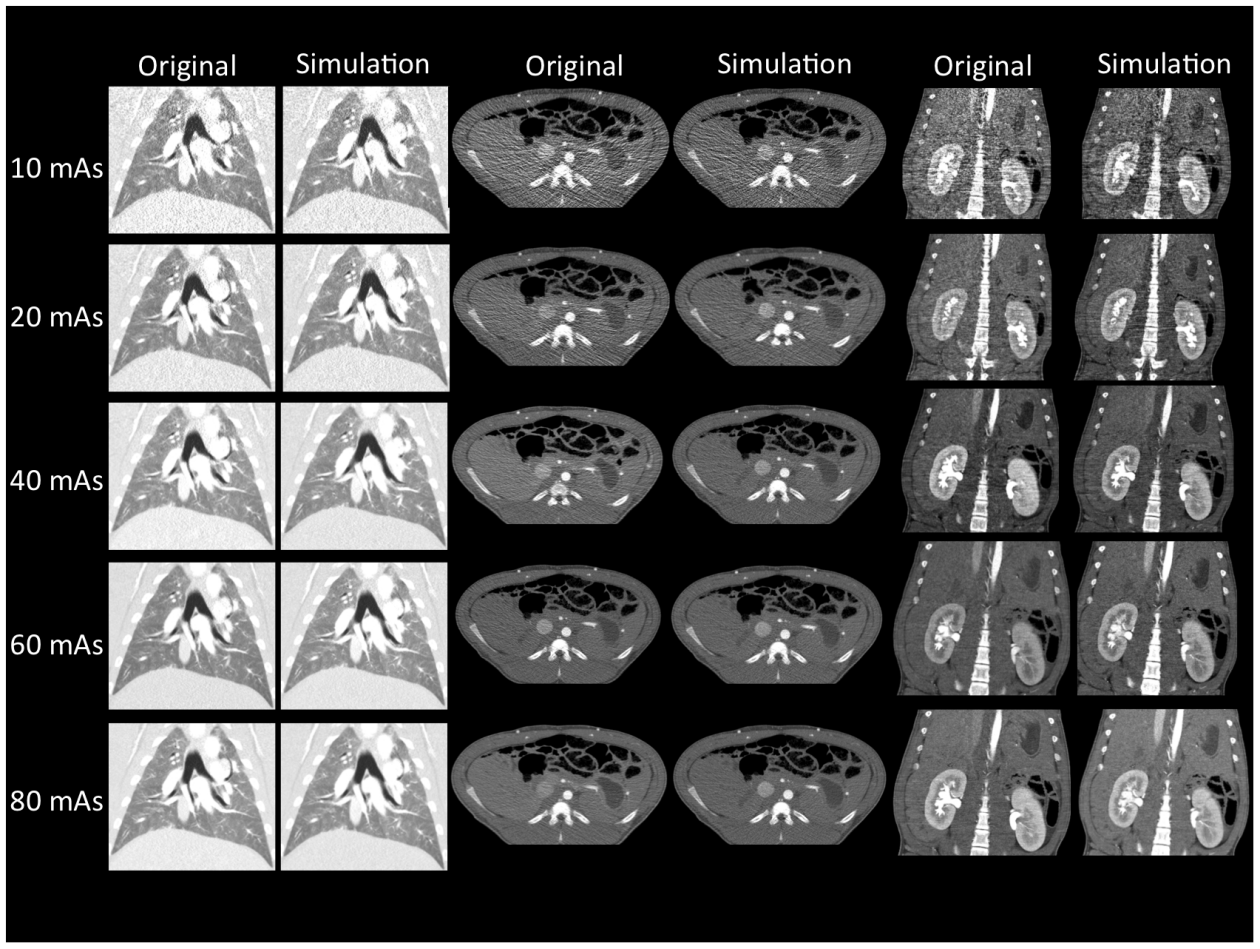
Coronal reformations with lung window settings (left side) showed similar image appearance of the lungs in simulated (right column) and original (left column) images. Simulated (right column) versus original (left column) images of the abdomen in transverse orientation are shown in the middle part. Image noise and streak artifacts are increased in lower dose images without visually detectable differences between original and simulated images. Also the simulated (right column) and original (left column) reformations of the abdomen including the kidneys in coronal orientation matched closely (right side).

Four radiologists rated a total of 640 images to be original or simulated. Figure 4 illustrates several examples of simulated and original images, presented for different tissue windows settings, and slice orientation. There is no visible difference between the originals and simulation. The total of 323 images (50.5%) were detected correctly as original (n = 160, 25%) or simulated (n = 163, 25.5%), p>0.05 respectively. The total of 317 images (49.5%) were mistaken to be an original (but simulated, n = 157, 24.5%) and to be a simulation (but original, n = 160, 25%). The detailed results for each observer are shown in Table 3. Comparing the results of all 4 observers, a total of 83 images were rated equally by at least 3 radiologists. In this regard, the group of radiologists consistently categorized 25 (29.1%) of the original images correctly as originals and 22 (25.6%) of the simulated images correctly as simulations. Beyond that, 45.3% (n = 39) of the images were congruently misclassified (original but simulation 24.4%, simulation but original 20.9%).

**Table 3:** Subjective image evaluation.

**observer 1**	**rated original**	**rated simulated**	**observer 2**	**rated original**	**rated simulated**
**Original**	37 (23.1%)	43 (26.9%)	**Original**	35 (21.9%)	45 (28.1%)
**Simulation**	42 (26.3%)	38 (23.8%)	**Simulation**	38 (23.8%)	42 (26.2%)
**observer 3**	**rated original**	**rated simulated**	**observer 4**	**rated original**	**rated simulated**
**Original**	42 (26.3%)	38 (23.8%)	**Original**	46 (28.8%)	34 (21.3%)
**Simulation**	39 (24.4%)	41 (25.6%)	**Simulation**	38 (23.8%)	42 (26.3%)

The total of 160 images (80 original and 80 simulated) were presented to four radiologists. The four possible combinations of image type (original or simulation) and image rating (original and simulation) showed similar percentages of about 25% (range 24.5%–28.8%), suggesting a subjective rating by random.

The four possible combinations of image type (original or simulation) and image rating (original and simulation) showed similar percentages of about 25% (range 24.5%–28.8%), without an observable discrimination between original and simulated images above chance level. Kappa values for all observer pairs were 0.06, −0.02, 0.09, 0.1, −0.08, and −0.09, respectively, which represents a poor interobserver agreement suggesting a subjective rating by random.

## Discussion

We showed in our study that it is possible to accurately simulate, based on a single acquired scan, another scan with lower dose than the actually acquired one. This technique offers the possibility to calculate lower dose images when only one examination was performed.

We approved congruent objective image parameters (noise level and HU-values) for a non-contrast CT examination of a swine. Prior studies concerning low dose computer simulation assessed metric parameters as noise and density values for comparison of original and calculated low dose images in digital chest radiography [7], tomosynthesis [8], and CT images [9]–[11]. Mayo et al. presented a computer modification tool for simulation images with increased image noise already in 1997 [12]. Prior simulation techniques mainly focused on the addition of image noise for simulation of lower dose images [10], [12], [13]. In our study, we evaluated a new technique [6], which makes use of the conditional variance identity to properly account for the variance of the input high-dose data, and allows for the inclusion of real samples of detector noise, properly scaled according to the level of the simulated x-ray signals. Phantom measurements using this technique and noise power spectrum analysis were described previously by Zabić et al. [6].

The major difference of our model compared the other models that we know of are the following: First, all other models make an approximation at some point in their derivation that a noisy signal from the high dose scan can be assumed noiseless. We use conditional variance identity to avoid that approximation which ultimately results in a method which does not depend on the noise variance in the original data set. We can start from any tube current and simulate any lower dose tube current without making that key approximation (described in detail in [6]). Another big step is that we use electronic noise samples from the real scanner, rather than simulating them as zero-mean Gaussian distribution. As discussed in [6] we conclude that if one wants to simulate contributions of the electronic noise correctly, then one has to take in account that the statistical distribution of the noise is strictly non-Gaussian.

We also included subjective evaluation of simulated images for contrast-enhanced CT in order to evaluate the potential of acquisition simulation in an examination setting closely adapted to the clinical examination of patients. This additional analysis was performed by a subjective image assessment by four experienced radiologists. They were not able do distinguish the original from simulated images above chance level, as the visual impact of original and simulated images was equivalent. This is of fundamental importance for the validation of a low dose simulator, as a final objective low dose simulation should be implemented in a clinical investigation setting. Here, radiologists will be able to determine the specific radiation dose that is necessary to achieve diagnostic quality of CT images by a minimized radiation exposure according to the ALARA principle.

CT is an essential imaging tool for the clinical day-to-day routine, as e.g. tumor follow-up in malignancy, trauma emergency department, or new techniques like perfusion imaging of the brain and the myocardium. There are a lot of CT examination protocols with different scan parameters (mAs, kVp, filters etc.) adapted to the individual clinical symptoms and specific diseases. On this note, with additional capabilities CT overs the number of protocols has significantly increased over the last years [14]. Especially the introduction of iterative reconstruction methods has widened the number of parameters possible for each protocol [15]–[20]. For each CT protocol, the optimal combination of required dose and imaging parameters has to be defined. However, this is problematic: systematic analysis of dose level and image quality would require repetitive scan of the patients, resulting in an inadequate high effective dose for those volunteers. Still, it is important to adjust the scan protocols to the standards of diagnostic imaging, while lowering the effective dose as far as possible. Adequate parameters for tube current and tube output have do be defined for each examination setting, but also for different scanners and different patient characteristics e.g. body weight. This topic is of special interest in pediatric radiology. Here, it is of special importance to define CT examination protocols providing diagnostic image quality by using preferably low radiation dose. Frush et al. presented a simulation technology for systematic evaluation of radiation dose reduction for abdominal multidetector CT of pediatric patients [21]. Thus, a valid dose simulation technique offers the possibility to perform dose calculation and optimization for CT examinations of each part of the body without repetitive scans of a group of test subjects.

In addition, low dose simulations can be used for education for medical technical assistants and the radiologists to depict the potential differences or equivalency of the same examination in the same patient at different dose levels. Training programs for radiological departments can help to substantially reduce radiation dose [22]. Therefore, CT simulation tools may visualize and facilitate the comprehension of potential dose saving strategies. Thus, the approach of dose reduction in routine clinical radiological examinations will attract increased interest, providing a concrete and demonstrative view on the resulting image quality and diagnostic value.

Lowering radiation dose is the hot topic of CT imaging techniques today. During the last ten years, several techniques for adapting of radiation dose to the patient physiognomy and the individual examination procedures were implemented to routine CT protocols [23]–[26]. In addition, there are several new approaches such as noise reduction techniques, iterative reconstruction or postprocessing techniques [15], [23], [27]–[29]. All these methods target a substantial decrease in radiation dose while maintaining diagnostic image quality. So routine CT examinations with effective radiation dose less than 1 mSv seem to be realistic in the near future. As a consequence, J. Thrall raised the question of considerations on radiation dose in clinical CT examinations should change from ALARA principle to AHARA (as high as reasonably achievable), pointing out the importance of a maximum benefit of diagnostic imaging using ionizing radiation dose [14]. So it will remain a challenge to optimize the balance between lowest radiation dose and highest diagnostic value. In this discussion, lower dose simulation techniques may help for visualization and determination of adequate dose settings in clinical CT.

There are some limitations of our study. First, the contrast enhancement of simulated and original images was not identical, because of slightly different contrast enhancement of the vessels and an accumulation of contrast material due to repetitive examinations; therefore, quantitative measurements have been performed on non contrast-enhanced scans. In addition, we did not use topogram-based tube current modulation in our study. However, as shown by Zabic et al., this simulation method is also compatible with tube modulation.

In conclusion, we showed that CT low dose simulation is a feasible and valid method for definition of adequate dose levels in CT. Thus, computer simulation of different dose levels provides an excellent base for future radiation dose optimization of diverse CT examination protocols for improved patient care.
